# Diagnostic Role of Radiology in Acute Gastrointestinal Bleeding: A Comprehensive Review

**DOI:** 10.7759/cureus.90219

**Published:** 2025-08-16

**Authors:** Maria Gabriela Cerdas, Rabah E R El Rayes, Ramkumar Kotehal, Sehrish Qaiser, Shamima Akther Rimpa, Shanida Rasheed, Shweta Menon, Sankar Ram Ragasankar, Humza F Siddiqui

**Affiliations:** 1 Medicine, Universidad de Ciencias Médicas (UCIMED), San Jose, CRI; 2 Emergency Medicine, Chesterfield Royal Hospital, Chesterfield, GBR; 3 General Internal Medicine, University Hospitals Birmingham NHS Foundation Trust, Birmingham, GBR; 4 Acute and General Medicine, University Hospitals Birmingham NHS Foundation Trust, Birmingham, GBR; 5 Internal Medicine, Medway NHS Foundation Trust, Kent, GBR; 6 Emergency Medicine, East Sussex Healthcare NHS Trust, Eastbourne, GBR; 7 General Medicine, Northern General Hospital, Sheffield, GBR; 8 Acute Medicine, University Hospitals Birmingham NHS Foundation Trust, Birmingham, GBR; 9 Internal Medicine, Jinnah Postgraduate Medical Center, Karachi, PAK

**Keywords:** acute gastrointestinal bleeding, ct angiography, git endoscopy, interventional radiology, lower gastrointestinal bleeding, magnetic resonance imaging, ultrasound, upper gastrointestinal bleeding

## Abstract

Acute gastrointestinal bleeding (GIB) is a major medical emergency with high morbidity and mortality. Endoscopy remains the first-line diagnostic and therapeutic approach, but radiological imaging has become increasingly important, particularly when endoscopy is inconclusive or unavailable. This review outlines the evolving role of radiological techniques, including computed tomography angiography (CTA), catheter-based angiography, radionuclide scintigraphy, magnetic resonance imaging (MRI), and ultrasound (US), in the detection, localization, and management of acute GIB. CTA is now the preferred imaging modality because of its speed, noninvasive nature, and superior anatomical detail. Catheter angiography not only enables precise diagnosis but also provides therapeutic options through embolization. Nuclear medicine techniques offer high sensitivity for intermittent or low-rate bleeding, while MRI and US contribute complementary insights in select patient groups. An integrated approach that combines radiological, endoscopic, and surgical strategies improves decision-making, shortens time to intervention, and enhances patient outcomes. Recent advances, including dual-energy CT, AI-assisted imaging, and novel embolic agents, are expected to further strengthen both diagnostic accuracy and therapeutic potential. Understanding the strengths, limitations, and sequencing of these modalities is key to optimizing care for patients with acute GIB.

## Introduction and background

Gastrointestinal bleeding (GIB) is a serious clinical concern, leading to significant morbidity and mortality if left untreated. It arises from diverse causes and is broadly categorized into upper and lower gastrointestinal tract sources. Upper gastrointestinal bleeding (UGIB) often results from peptic ulcers, esophageal varices, Mallory-Weiss tears, or gastric neoplasms, and typically presents with hematemesis or melena. Lower gastrointestinal bleeding (LGIB), more commonly due to diverticular disease, colorectal malignancies, inflammatory bowel disease (IBD), or hemorrhoids, usually manifests as hematochezia [1].

UGIB poses a particular clinical challenge, accounting for about 75% of all acute gastrointestinal hemorrhages. Its annual global incidence ranges from 48 to 160 per 100,000 individuals (0.05%-1%), with men more commonly affected [1]. Although peptic ulcer bleeding resolves spontaneously in up to 80% of cases [2], UGIB remains associated with higher severity, including a sixfold greater likelihood of hospitalization and mortality rates between 2% and 15% compared with LGIB [3]. By contrast, LGIB has an incidence of approximately 87 per 100,000 individuals. Most episodes (about 85%) resolve without intervention, but rebleeding occurs in up to 38% within a year, one-quarter require transfusion, and mortality can reach 3.9% depending on bleeding source and comorbidities. These figures underscore the urgent need for effective hemostatic strategies and ongoing research to optimize clinical outcomes [4,5].

**Figure 1:**
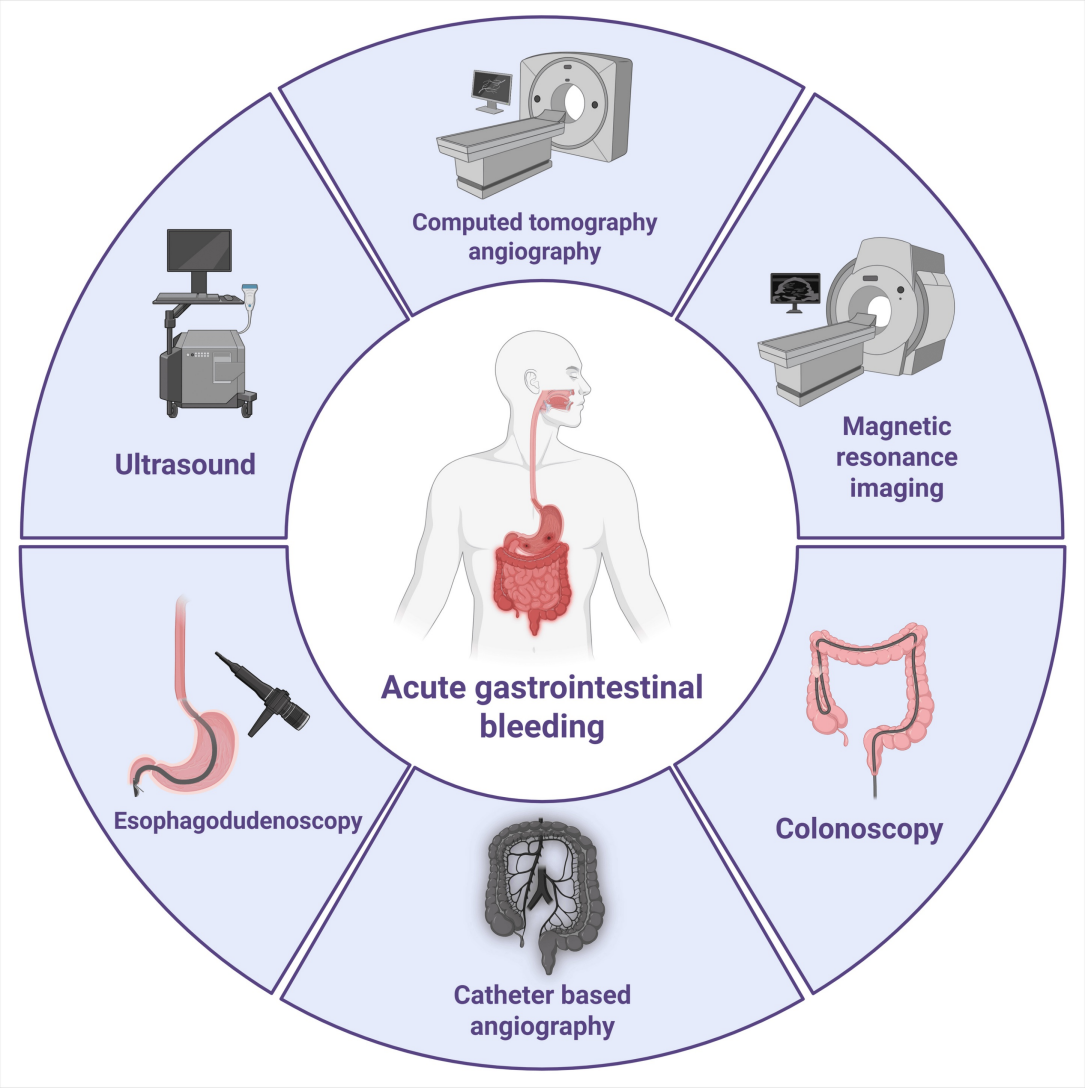
Summary of radiological modalities used in the diagnosis of gastrointestinal bleeding. Image Credit: Humza Siddiqui. This image was created with BioRender.com.

The diagnostic and therapeutic landscape for GIB has advanced significantly with developments in radiological imaging. Computed tomography angiography (CTA) is now a preferred noninvasive modality, particularly when endoscopy is inconclusive, as it provides rapid localization and detailed vascular mapping to guide interventions. Catheter-based angiography remains critical for both diagnosis and treatment through targeted embolization. Nuclear scintigraphy also retains a role in detecting intermittent or occult bleeding when other imaging is nondiagnostic [6]. Transcatheter arterial embolization (TAE), including the use of cyanoacrylate glue, has become an essential therapy for acute non-variceal UGIB, particularly after failed endoscopic intervention. However, access to TAE is often limited to tertiary centers due to its requirement for specialized expertise and infrastructure [7,8]. Evidence further supports prophylactic TAE in select patients to reduce rebleeding risk, underscoring its expanding role in severe gastrointestinal hemorrhage [9]. Risk stratification tools, such as the Rockall score (RS), aid in assessing prognosis, while embolization techniques provide effective hemorrhage control by occluding culprit vessels [7,10]. Figure 1 summarizes the radiological modalities used in diagnosing and managing GIB.

In this narrative review, we provide a comprehensive overview of the role of radiology in diagnosing acute GIB, aiming to aid clinicians and radiologists in developing diagnostic guidelines that enable prompt and accurate evaluation, ultimately improving patient outcomes and clinical care.

## Review

Types and etiologies of GIB

GIB refers to any bleeding that occurs in the gastrointestinal tract, from the mouth to the anus. It is broadly classified into two categories: UGIB and LGIB. The ligament of Treitz serves as the anatomical landmark distinguishing the two; bleeds proximal to the ligament are classified as UGIB, while those distal are considered LGIB. This division is clinically important, as it guides both evaluation and treatment [11,12]. 

UGIB can be acute or chronic, slow or brisk, and either obscure or overt, depending on the underlying cause, rate, and duration of blood loss [12]. Peptic ulcer disease (PUD) is the most common etiology, accounting for nearly half of hospital admissions [13]. *Helicobacter pylori* infection and the use of non-steroidal anti-inflammatory drugs (NSAIDs) contribute to about 80% of PUD-related bleeding [14]. Other frequent causes include esophagitis, gastric erosions, Mallory-Weiss tears, Dieulafoy lesions, varices, and neoplasms such as gastric cancers [15]. The clinical presentation of UGIB is variable but often includes hematemesis and melena. Hematemesis refers to the vomiting of fresh blood or clots, while melena describes black, sticky, tarry stools with a characteristic odor. "Coffee-ground" vomiting denotes the presence of dark specks of partially digested blood in vomitus. Although more typical of LGIB, hematochezia, passage of bright red blood per rectum, can also occur in cases of brisk UGIB [14,15].

Severe LGIB is more common in older patients and men. Its causes can be broadly categorized as vascular, inflammatory, neoplastic, traumatic, or iatrogenic. Common etiologies include diverticular disease, angiodysplasia, inflammatory bowel disease (IBD) such as Crohn’s disease and ulcerative colitis, colorectal neoplasms (including cancer), and benign anorectal conditions like hemorrhoids, anal fissures, and rectal ulcers [16]. Diagnosis can be particularly challenging in colonic diverticular bleeding, which accounts for about 25%-65% of LGIB cases. Nearly 80% of diverticular bleeds resolve spontaneously, making localization of the culprit vessel difficult. Rebleeding occurs in roughly 20%-38% of cases, further underscoring the importance and difficulty of identifying the bleeding source [17].

LGIB is generally classified into three types: massive, moderate, and occult bleeding. Massive bleeding usually affects patients over 65 years with multiple comorbidities. It typically presents with hematochezia and hemodynamic instability, most often caused by diverticulosis or angiodysplasia, with mortality rates reaching up to 21% [12]. Moderate LGIB can occur at any age and usually presents with hematochezia or melena in otherwise stable patients. Causes include inflammatory, infectious, or neoplastic conditions, as well as benign or congenital anorectal disorders [18]. Occult LGIB may present at any age and is often diagnosed incidentally on laboratory testing, typically revealing microcytic hypochromic anemia from chronic blood loss. These patients are generally stable, and common causes include neoplastic, inflammatory, and congenital conditions [19].

Diagnostic modalities

Endoscopy

Esophagogastroduodenoscopy (EGD): EGD is considered the gold standard for diagnosing UGIB, with a reported sensitivity of 92%-98% and specificity ranging from 30%-100% [20]. It allows direct visualization from the esophagus to the proximal duodenum and serves both diagnostic and therapeutic purposes. Interventions include local drug administration, thermal or electrocautery coagulation, and placement of hemostatic clips to achieve hemostasis [21]. However, potential complications include perforation and procedure-related bleeding [19].

Early endoscopy is central to risk stratification in UGIB, helping identify low-risk patients who can be discharged early and high-risk patients requiring urgent treatment. Current guidelines recommend performing EGD within 24 hours of presentation. Studies show no significant difference in outcomes between procedures done within 12 hours and those performed between 12 and 24 hours [20]. However, some studies have questioned whether performing endoscopy within six hours (urgent endoscopy) leads to better outcomes than the conventional 6-24-hour time frame. Laursen et al. found that endoscopy performed within 6-24 hours reduced mortality in hemodynamically unstable patients with peptic ulcer bleeding, while stable patients with high American Society of Anesthesiologists (ASA) scores (3-5) had better outcomes when endoscopy was performed within 12-36 hours [22]. Similarly, Nukala et al., in a cohort of 40 patients with acute UGIB, reported that endoscopy identified the bleeding source in 97.5% of cases and frequently provided definitive therapy, especially in variceal banding [23].

Colonoscopy

Colonoscopy, following adequate colonic purge, remains the primary diagnostic and therapeutic procedure for most patients with LGIB once hemodynamic stability is achieved [24,25]. It allows direct visualization, identification of bleeding sources in 45%-90% of cases, and therapeutic interventions such as endoscopic hemostasis [24]. Importantly, colonoscopy can identify stigmata of recent hemorrhage (SRH), which strongly predicts rebleeding risk. A prospective study reported a 66% rebleeding rate within 30 days in patients with SRH when no endoscopic therapy was provided, whereas no rebleeding occurred in patients without SRH [26]. Limitations include the need for bowel preparation to optimize visualization and reduce perforation risk, as well as sedation requirements, which may be unsafe in unstable patients [24,27].

The timing of colonoscopy in LGIB remains debated. A multicenter retrospective cohort study compared early colonoscopy (EC) (within 24 hours), elective colonoscopy (24-48 hours), and delayed colonoscopy (DC) (48-120 hours). EC was associated with earlier detection of significant rebleeding and shorter hospital stays but also with a higher risk of rebleeding, without improvement in mortality, need for interventional radiology, or surgical intervention [28]. Current evidence suggests that early colonoscopy may be most useful in high-risk patients, such as those with a shock index (SI) ≥ 1 or a performance status ≥3 at presentation [28].

Radiological modalities in acute GIB

Radiological imaging plays a crucial role in both the diagnosis and management of GIB. Commonly used techniques include computed tomography angiography (CTA), catheter-based angiography, ultrasound (US), and magnetic resonance imaging (MRI). Nuclear medicine studies are also valuable in detecting active bleeding, localizing the source, assessing severity, and guiding therapeutic interventions.

Computed Tomography Angiography (CTA)

CTA is a key imaging modality for diagnosing acute GIB, with reported sensitivity of 79%-95% and specificity of 95%-100% [29]. Its diagnostic accuracy is comparable to scintigraphy, but CTA is faster, more widely available, and feasible in emergency settings. The bleeding detection threshold of CTA (0.3-0.5 mL/min) is also lower than that of angiography, which is mainly reserved for therapeutic interventions [30].

In hemodynamically unstable patients, CTA is often preferred over colonoscopy because it rapidly identifies bleeding sources in the upper GI tract or small bowel, requires no bowel preparation, and is broadly accessible. However, in patients with rectal bleeding and instability, upper GI endoscopy should still be considered before CTA. For optimal vascular opacification, IV contrast is administered at 4-5 mL/s, and active bleeding appears as contrast extravasation with pooling on delayed images [31]. CTA is non-invasive, fast, and widely available, but it has limitations, including radiation exposure, inability to provide immediate therapy, and difficulty in detecting very slow or intermittent bleeding. Still, it provides important preoperative insights for embolization, surgery, or endoscopic intervention, and it can be used to assess unexplained rebleeding after prior treatment [29]. Advances in CT technology have led to the creation of dual-energy CTA (DECTA), which scans tissues at different kilovoltages. DECTA enhances contrast resolution, reduces artifacts (e.g., from metallic implants), improves localization of bleeding sites, and allows lower radiation and contrast doses [30].

Clinical studies further highlight CTA’s role. Nagata et al. compared colonoscopy alone with CT followed by colonoscopy in acute LGIB and found CT improved vascular lesion detection (35.7% vs. 20.6%) and increased therapeutic endoscopy (34.9% vs. 13.4%) [32]. Reported sensitivity and specificity were 37.8% and 88.9% for vascular lesions, and 81.3% and 80.9% for inflammation or tumors, providing an overall 15% diagnostic benefit [32]. Similarly, Clerc et al. compared CTA with lower endoscopy (LE) and found CTA was performed more quickly (3 vs. 22 hours) and identified active bleeding more often (31% vs. 15%). The study concluded that CTA effectively localizes bleeding and helps predict surgical need [33].

Catheter-Based Angiography

Catheter angiography is both a diagnostic and therapeutic tool in acute GIB, particularly when initial imaging is inconclusive or after a positive CTA. Prompt intervention is critical. Studies show that performing angiography within 90 minutes of a positive CTA increases the likelihood of detecting active bleeding by up to eightfold [24]. Diagnostic accuracy varies, with reported sensitivity averaging 60% and specificity approaching 100% [20]. Success rates for embolization range from 73%-100% in LGIB and 60%-100% in UGIB [34]. Angiography can detect bleeding rates as low as 0.5 mL/min and can also identify non-bleeding causes, such as tumors or vascular malformations.

A major advantage of angiography is its ability to provide immediate treatment. Active bleeding is identified by contrast extravasation into the bowel lumen or wall, often with pooling on delayed angiograms. Once localized, selective embolization can be performed, making it especially valuable in patients at high surgical risk or when endoscopic treatment fails. However, angiography has limitations: it is invasive, may miss intermittent bleeding, and carries risks such as bowel ischemia, vessel injury, and contrast-related complications [10,30].

Ultrasound (US)

Ultrasound has a limited role in the direct diagnosis of GIB but can provide important complementary information. It is particularly useful in detecting underlying colonic conditions associated with bleeding, such as diverticulosis, colonic neoplasms, or IBD, and is often employed for image-guided interventions [35]. Limitations include operator dependence, artifacts, and incomplete visualization of the gastrointestinal tract [36].

Advanced ultrasound techniques expand its utility. Doppler US (DUS) can assess vascular abnormalities, while contrast-enhanced US (CEUS) improves the detection of active bleeding by using microbubble contrast agents that remain intravascular, distinguishing blood flow from surrounding tissue. CEUS offers several advantages: it does not require prior lab tests, has a strong safety profile, and carries few contraindications [37]. Endoscopic ultrasound (EUS) provides access to abdominal vessels and is a valuable option in patients with persistent bleeding refractory to conventional treatments. EUS can accurately localize the bleeding source, characterize its features, and guide targeted therapies [38]. Emerging evidence highlights the role of point-of-care ultrasound (POCUS) in risk stratification. A study by Chen et al. found that POCUS enhanced the predictive accuracy of Rockall, Glasgow-Blatchford, and Velayos scores for complications and outcomes in GIB, improving early detection of adverse events (AEs) in LGIB and late AEs in UGIB [39]. Similarly, a recent case series reported that POCUS helped predict aspiration risk, confirm diagnosis, and guide early goal-directed management in UGIB patients [40].

Magnetic Resonance Imaging (MRI)

MRI is the most advanced imaging modality, providing high-resolution cross-sectional views without ionizing radiation and with excellent soft-tissue differentiation. It is particularly useful in pediatric and pregnant patients, where radiation exposure from CT is a concern [41,42]. Magnetic resonance enterography (MRE) plays a complementary role to colonoscopy in symptomatic patients with colonic IBD [42]. MRE aids diagnosis, staging, and monitoring of disease activity using validated scoring systems such as the Magnetic Resonance Index of Activity (MaRIA) and the Magnetic Resonance Enterography Global Score (MEGS). It is especially valuable in assessing Crohn’s disease, including transmural healing, which is considered the most reliable marker of treatment response [43-45]. MRI can also help differentiate Crohn’s disease from ulcerative colitis when endoscopy is incomplete or contraindicated [46].

MRI is effective in evaluating diverticulitis, with reported sensitivities of 86%-94% and specificities of 88%-92%. Findings such as inflamed pericolic fat, thickened diverticula, mesenteric infiltration, and segmental stenosis can be reliably detected [47,48]. Additionally, MRI may aid in assessing obscure bleeding in younger patients, particularly from small bowel neoplasms [49]. Despite these advantages, MRI has limitations, including motion artifacts from bowel peristalsis or patient movement, longer scan times, and the need for oral contrast. Its role in emergent GIB remains limited, though experimental approaches such as real-time multi-contrast magnetic particle imaging (MPI) show promise in overcoming motion artifacts [50].

Radionuclide Imaging

Technetium-99m (Tc-99m) labeled red blood cell (RBC) scintigraphy is a sensitive tool for detecting gastrointestinal bleeding due to the tracer’s long intravascular half-life, which permits monitoring over several hours. A study is considered positive if a new focus of extravascular activity appears, increases in intensity over time, and moves in an anterograde or retrograde fashion consistent with bowel anatomy [51].

Its greatest advantage is sensitivity, as it can detect bleeding rates as low as 0.05-0.10 mL/min, making it one of the most sensitive modalities available [41]. Other benefits include being noninvasive, not requiring iodinated contrast or bowel preparation, and its ability to detect both arterial and venous bleeding. However, the technique has important limitations. It is best suited for hemodynamically stable patients, as it requires prolonged imaging. Accurate localization of the bleeding source can be difficult, with mislocalization reported in 10%-30% of cases, although integration with SPECT/CT improves accuracy [41]. Compared with CT angiography, which is preferred in unstable patients with active bleeding, radionuclide imaging is generally reserved for low- or intermittent-rate bleeding in stable patients [41]. Scintigraphy still has a defined role in the evaluation of Meckel’s diverticulum, particularly in children. Tc-99m pertechnetate highlights ectopic gastric mucosa, typically seen as a focal area of uptake in the right lower quadrant, appearing simultaneously with gastric activity [52]. A meta-analysis in pediatric patients reported a pooled sensitivity of 0.80 and high specificity, supporting its diagnostic value in this setting [53].

Interventional radiology (IR)

Interventional radiology has become a cornerstone in managing UGIB and LGIB refractory to endoscopy [54]. Its popularity has increased due to its minimally invasive nature and favorable safety profile, particularly in elderly and high-risk patients when compared with surgical options [55,56]. TAE involves angiographic identification of the bleeding vessel, followed by embolization using hemostatic materials to achieve hemostasis [57,58]. It is traditionally employed in UGIB when endoscopy fails or when rebleeding occurs after endoscopic intervention [58,59]. Other candidates include patients in whom no bleeding site is identified during endoscopy or those with bleeding in endoscopically inaccessible sites, particularly within the small intestine [54,59]. It is also considered superior in the management of acute pancreatic bleeding, as well as vascular abnormalities such as Dieulafoy lesions, which are often difficult to visualize [60]. Hemobilia, a rare cause of UGIB, can also be effectively managed by this interventional procedure [61]. TAE is additionally used as a first-line option in hemodynamically unstable patients with LGIB unresponsive to conservative management [3,62]. A pre-procedure CT angiography is usually considered a prerequisite before TAE to identify the bleeding source [55]. Correction of any coagulopathy is also advised, if possible, before the procedure to optimize its effectiveness [58].

The TAE is conventionally performed using transfemoral access. A super-selective catheterization technique with co-axial and/or tri-axial microcatheters is employed to access the suspected bleeding vessel for embolization [58,63]. This approach reduces the risk of non-target embolization and ischemic events [57]. Recently, increasing evidence has supported empiric embolization in the management of UGIB based on endoscopic findings, even in the absence of a positive angiographic result. This has shown comparable therapeutic efficacy to targeted embolization in terms of rebleeding rates and post-embolization survival [62,64]. Currently, multiple embolic agents are available, which may be used individually or in combination [62,65]. The selection of an embolic agent is multifactorial, depending on the cause of bleeding, the interventionist’s preference, and local availability [54,63].

The clinical success of TAE is generally defined by the prevention of rebleeding and early mortality [66], while technical success refers to procedural accuracy and the ability to achieve acute hemostasis [62]. A retrospective observational study of 59 patients who underwent TAE for acute GIB reported a technical success of 100% [67]. Similarly, a recent retrospective study in 266 patients with UGIB found a technical success rate of 97.3% and a clinical success rate of 73.1% [68]. In a multicenter study, technical and clinical success rates of 100% and 93.8%, respectively, were observed in 128 patients with LGIB treated with TAE [69]. The variability in clinical outcomes is often influenced by underlying etiology, coagulopathies, and comorbidities [55,70]. With its increasing role in acute GIB, the potential for prophylactic embolization, particularly for bleeding ulcers, appears promising [3,71]. However, complications can occur. Vascular site hematomas are among the most common, while pseudoaneurysms may present with pain and swelling, with management depending on size [59]. Bowel, hepatic, or splenic ischemia represents major risks directly related to embolization, especially with glue use. Contrast-induced nephropathy is another important consideration [62,71].

Diagnostic protocol for GIB

A detailed history and physical examination are crucial to identify potential sources of GIB and to assess severity, risk of rebleeding, and mortality. Key factors to consider include underlying conditions such as cardiovascular and renal disease, a history of gastrointestinal surgeries, and symptoms like abdominal pain, altered bowel habits, or unexplained weight loss. Reviewing medications is also essential, particularly NSAIDs, antiplatelets, and anticoagulants. Hematochezia may occasionally result from massive UGIB, especially in patients with hemodynamic instability or risk factors such as portal hypertension or a history of PUD. Physical examination should prioritize vital signs and signs of hypovolemia. The presence of melena on digital rectal examination suggests an upper GI source, while evidence of ongoing bleeding on rectal exam, especially in elderly patients with significant comorbidities and hemodynamic instability, strongly influences the prognosis of LGIB [72].

There are several risk stratification scores available for patients presenting with GIB. For UGIB, the most commonly used tools are the Glasgow Blatchford Score (GBS), RS, and AIMS65 (albumin, INR, mental status, systolic blood pressure, age >65 years). A GBS ≥ 2 is typically used as a threshold for hospitalization. For LGIB, widely recommended scores include the Oakland, SHA(2)PE, and NOBLADS (NSAID use, no diarrhea, no abdominal tenderness, blood pressure ≤ 100 mmHg, antiplatelet use (non-aspirin), disease score ≥ 2, and syncope). An Oakland score ≤ 8 identifies low-risk patients suitable for safe discharge, whereas a score >8 warrants admission. Despite their availability, the utility of these scores is limited. While prognostic scores may help identify LGIB patients at risk for severe bleeding and poor outcomes, their role in guiding management and improving outcomes remains unproven [73]. Currently, hemodynamic status is the primary determinant of whether urgent CTA or non-urgent colonoscopy is needed in admitted LGIB patients [10,11,72]. Despite these advancements, no single score has been widely accepted for predicting adverse outcomes among patients presenting with GIB. However, to guide CTA use in unstable patients, a SI > 1 is considered clinically significant for all major overt bleeds [17,74-77]. Urgent upper endoscopy within 12 to 24 hours should be considered among unstable patients with UGIB with suspected variceal bleeding [78]. For UGIB, urgent endoscopy within 12-24 hours is recommended in unstable patients, particularly when variceal bleeding is suspected [78]. In cases of acute unstable GIB, CTA is the preferred diagnostic modality. Patients with SI < 1 are generally stable and may undergo colonoscopy during hospitalization or as an outpatient, with concurrent evaluation for possible UGIB sources if indicated. For admitted LGIB patients, colonoscopy remains the primary diagnostic tool, allowing for bleeding site identification, biopsies, and therapeutic intervention when appropriate [79-86]. Evidence on the timing of colonoscopy remains mixed. A nationwide analysis of LGIB patients compared EC (<24 hours) with DC (>24 hours) and found that EC reduced inpatient mortality (0.9% vs. 1.4%, p < 0.001) and shortened hospital stay (3 vs. 5 days, p < 0.001). However, inverse probability treatment weighting showed these differences were not significant [87]. Similarly, a meta-analysis of randomized trials reported that EC within 24 hours did not significantly reduce mortality or rebleeding compared with elective colonoscopy after 24 hours [88]. Diagnostic protocols for GIB are summarized in Tables 1-4.

**Table 1:** Protocols for hemodynamic instability CTA: computed tomography angiography, UGIB: upper gastrointestinal bleeding, LGIB: lower gastrointestinal bleeding, EGD: esophagogastroduodenoscopy. Source: [29,76-78].

Aspect	British guidelines	European guidelines	American guidelines
Definition	Shock Index ≥ 1 = instability	Shock Index ≥ 1 = instability	Defined by hypotension, tachycardia, or ongoing blood loss
Initial Imaging	CTA first-line, followed by angiography or endoscopy	CTA preferred for localization	CTA preferred in unstable patients or those with ongoing bleeding
UGIB rule-out	Upper endoscopy if CTA is negative or UGIB suspected	Gastroscopy unless CTA confirms LGIB	EGD within 24 hours in unstable patients or when UGIB suspected; urgent (<12h) if variceal bleeding suspected

**Table 2:** Imaging and diagnostic protocol CTA: computed tomography angiography, Tc-99m RBC: technetium-99m labeled red blood cell, VCE: video capsule endoscopy, GIB: gastrointestinal bleeding. Source: [79,80].

Aspect	British guidelines	European guidelines	American guidelines
CTA	79%-95% sensitivity, 95%-100% specificity; fast and accessible	Same stats; preferred over RBC scintigraphy	First-line imaging; fast and widely available
Tc-99m RBC scintigraphy	Used if CTA is inconclusive or an intermittent bleeding is suspected	Similar, but less favored than CTA	Considered if CTA unavailable or intermittent bleeding suspected
VCE	Use after negative endoscopy; best if within 48 hours	Not emphasized	Used for obscure GIB after negative scopes

**Table 3:** Protocols for colonoscopy RCTs: randomized clinical trials, CTA: computed tomography angiography. Source: [24,80-82].

Aspect	British guidelines	European guidelines	American guidelines
Role	First line in stable patients. Diagnostic yield up to 90%	First line in stable. Offers both diagnosis and therapy	Recommended during the same hospitalization
Urgency	Early (<24h) vs. delayed: mixed evidence; may increase rebleeding	RCTs: no clear outcome difference between early and elective colonoscopy	Early colonoscopy slightly improves inpatient mortality and length of hospital stay, although data stratification may yield some outcomes statistically insignificant
Prep consideration	Requires adequate bowel preparation.	Requires adequate bowel preparation. CTA is preferred if unstable due to no prep needed	Colonoscopy may be deferred until stable and prepped

**Table 4:** Protocols for angiography and embolization CTA: computed tomography angiography. Source: [83-88].

Aspect	British guidelines	European guidelines	American guidelines
When	If CTA is positive and bleeding is ongoing	Preferably within 60-90 minutes of positive CTA	After CTA, if bleeding is ongoing or endoscopy fails
Rebleed risk	10%-50% short-term; 25% long-term	0%-50% depending on source; ischemia risk 1%-4%	Rebleeding possible; low ischemia risk with super-selective embolization
Use	Reserved for severe bleeds not controlled by endoscopy	Reserved for severe bleeds not controlled by endoscopy	Strong recommendation if active extravasation on CTA or failed endoscopy

## Conclusions

Radiological imaging is essential in the diagnosis of acute GIB, particularly in emergency settings where conventional endoscopy may be unsafe or inconclusive in hemodynamically unstable patients. CTA is now the preferred non-invasive modality, allowing rapid and accurate localization of active bleeding. Catheter angiography provides both diagnostic and therapeutic options, especially in unstable or refractory cases. Nuclear medicine and MRI serve as useful alternatives in select situations, such as intermittent bleeding or small bowel disease. Emerging modalities are focused on improving diagnostic accuracy, minimizing radiation exposure, and enhancing procedural outcomes. Large randomized controlled trials are needed to establish reliable decision-making algorithms and optimize patient outcomes. Developing standardized guidelines across institutions is imperative to support timely diagnosis and effective management of acute GIB.
